# Health Disparities from Economic Burden of Diabetes in Middle-income Countries: Evidence from México

**DOI:** 10.1371/journal.pone.0068443

**Published:** 2013-07-12

**Authors:** Armando Arredondo, Gabriela Reyes

**Affiliations:** Research Center on Health System, National Institute of Public Health, Cuernavaca, Morelos, México; University of Texas Health Science Center at San Antonio, United States of America

## Abstract

The rapid growth of diabetes in middle-income countries is generating disparities in global health. In this context we conducted a study to quantify the health disparities from the economic burden of diabetes in México. Evaluative research based on a longitudinal design, using cost methodology by instrumentation. For the estimation of epidemiological changes during the 2010–2012 period, several probabilistic models were developed using the Box-Jenkins technique. The financial requirements were obtained from expected case management costs by disease and the application of an econometric adjustment factor to control the effects of inflation. Comparing the economic impact in 2010 versus 2012 (p<0.05), there was a 33% increase in financial requirements. The total amount for diabetes in 2011 (US dollars) was $7.7 billion. It includes $3.4 billion in direct costs and $4.3 in indirect costs. The total direct costs were $.4 billion to the Ministry of Health (SSA), serving the uninsured population; $1.2 to the institutions serving the insured population (Mexican Institute for Social Security–IMSS-, and Institute for Social Security and Services for State Workers-ISSSTE-); $1.8 to users; and $.1 to Private Health Insurance (PHI). If the risk factors and the different health care models remain as they currently are in the analyzed institutions, health disparities in terms of financial implications will have the greatest impact on users’ pockets. In middle-income countries, health disparities generated by the economic burden of diabetes is one of the main reasons for catastrophic health expenditure. Health disparities generated by the economic burden of diabetes suggests the need to design and review the current organization of health systems and the relevance of moving from biomedical models and curative health care to preventive and socio-medical models to meet expected challenges from diseases like diabetes in middle-income countries.

## Introduction

Diabetes is a health problem that requires a comprehensive approach, since its tendency to increase has not been addressed and economic resources have not been assigned for its resolution. The high costs in health and Mexico’s demographic behavior, in which a change in the population pyramid is noticed, will add risks for the adult population, and increase the demand for health services in the near future [1]–[3]. In this context diabetes and its complications are a great economic challenge for any health system, particularly when it is present in older adults; this is because of the high prevalence of complications in this type of population, in any society [4].

Mexico occupies the ninth place world-wide in diabetes prevalence. This is a truly alarming fact, but projections of international specialists show that for the year 2025, the country will occupy the seventh place in prevalence of this disease. Diabetes has become a world-wide epidemic due to the high death rates that have been registered in the last 10 years. For example, in Mexico one third of heart attacks and half of all chronic renal failures are direct consequences of diabetes [5]–[6]. In this context, the diverse health institutions in the country have begun to reinforce their preventive campaigns to avoid even higher costs. Indeed, by the time the diagnosis of diabetes is made, and of its complications, the costs of treatment are already very high and the patient is gradually losing his/her productive life-years, with important repercussions in terms of indirect costs [7]–[9]; in addition, the costs to the quality of life of these patients are very high. The financial burden that is put on the health sector to control the problems associated with diabetes is not known in most countries, making it difficult to make an efficient allocation of resources and to implement strategic planning to face the demand for health services related to this disease [10]–[12].

In Mexico, these tendencies have been widely documented by the National Survey of Chronic Diseases and in findings of the 2006 Health and Nutrition National Survey [13]. For example, diabetes mellitus prevalence has increased from 4.6% in 1993, to 22.8 in 2006. In relative terms, its proportional contribution to the mortality of the country went from 0.4 percent to more than ten percent, thus becoming the third cause of death in Mexico [14]–[16]. The future perspective indicates that the increase in the amount of diabetes and its complications will continue [17]. The impact of this disease is not only on mortality but also on morbidity and the quality of life, since it represents an enormous burden for the individual and his/her family, as well as for the health system and society in general.

In addition to the epidemiological background of diabetes, the increase in the demand for health services and in health care costs for this type of patients is also noteworthy. Indeed, it is quite evident that the increase in costs of health services and, hence, in health expenditures and changes in modes of resource allocation for diabetes, make it imperative to incorporate the economic perspective in the analysis of the health sector [18]–[19].

In this context, the use of economic indicators, adds new elements to make decisions on the allocation of resources to ensure universal coverage of care for diabetic patients. [20]. In economic terms, the changes in the epidemiologic and demographic profiles mean an increase in the demand for health care for expensive diseases (treatment of chronic degenerative diseases and accidents) that will compete with the budget assigned for the treatment of infectious diseases. Thus, it is important to remember that there will be a need to reassess health priorities and to establish the strategic actions that will allow for optimal use and organization of health resources for diabetes mellitus [21]–[22].

In summary, the epidemiological changes of diabetes and the constant increases in the cost of care are a major public health challenge that is being faced in most countries in the world, but particularly in Mexico where health financing is characterized by a highly fragmented health system with high costs for everyone, including patients and society. In this sense, the purpose of this paper is to identify the economic burden of diabetes and its implications for the health system, patients and society.

## Methods

Evaluative research based on a longitudinal design to determine the 2010–2012 financial requirements to deliver health care for diabetes type 2. The studied institutions were SSA, IMSS and ISSSTE. The annual demand for health care services for diabetes was calculated from the number of cases being treated, adjusted by type of institution. The population base of the study included the registration of 4,854,689 patients with medically diagnosed with diabetes mellitus in years prior to the study. This information was obtained from the statistics bulletin on health impairment of the National Health System [13]; [17].

Case management was defined for an average case with the corresponding adjustments for each institution. For each service and event to be evaluated, management of the average case was defined on the basis of the disease’s natural history and the results of shadow study reviews. The point of view of a group of expert clinicians and administrators was considered in order to obtain a homogeneous opinion of how to manage average case. These definitions refer to the demand of hospital or ambulatory services, according to diabetes care.

As explained above, standard management was defined per service/event to be cost-evaluated, considering the natural history of the disease, results found in the clinical files review, the shadow study, and the opinions of a group of expert clinicians and administrators. Results obtained depended on hospital or ambulatory management, the case and the institution’s regulations.

Direct costs of health care services were obtained from the management of standardized cases, adjusted by type of institution. The cost-evaluation method was designed according to an instrumentation technique which identified production and supply functions for each case management. Eight tools were used to establish costs per production function, which were concentrated in cost-evaluation matrices, according to the health providers. These matrices concentrated costs of inputs (human resources, material resources, infrastructure, drugs, laboratory tests, etc.) and average costs for annual case management from the following production functions:

− Medical consultation for first time

− Medical visit for check control subsequent

− Laboratory studies

− Hospitalization for diabetic decompensation

− Intermediate therapy

− Complications

− Concentrate input costs

− Concentrate costs of average case

It is important to note that input costs were obtained from the catalog of salaries for health human resources and purchasing catalog for health supplies in Mexico [23].

Indirect costs were obtained following the human capital approach to calculate indirect costs of chronic diseases in Latin America [24]–[25]. Models were designed to include three categories of monetary costs attributable to diabetes in three public institutions: mortality costs, cost of permanently disabled patients and cost of temporarily disabled patients.

To estimate premature mortality costs, we calculated the indirect cost of premature mortality due to diabetes. We used the number of premature deaths by diabetes of the general mortality record of vital statistics, by type of institution [26]. Costs of premature mortality were obtained multiplying the total annual lost life years accumulated in those under 65 years, multiplied by the per capita GNP. Like this, we obtained the total indirect costs attributable to premature mortality. To estimate permanent disability, the “years of productive life lost” indicator was used, multiplied by the per capita GNP for 2011. Temporary disability costs were obtained using disability indicators on chronic diseases from the National Health Survey for Chronic Diseases in Mexico [13]. For each institution, the mean number of disability days related to diabetes was used, multiplied by the number of temporary disabilities estimated for 2011; this figure was multiplied by the daily cost of lost productivity as a function of the per capita GNP for 2011 [27].

In order to determine the financial requirements of the 2010–2012 period, a time series study was performed for the 1989–2009 period with a probabilistic design according to the Box-Jenkins technique [28], using a confidence interval of 95% with P<.05. Analysis variables included changes in morbidity for the study period and changes in health policies and programs for diabetes and chronic diseases. The following steps were taken in the development of the probabilistic models:

Step 1. Identification: a) identifying the tentative model for the time series for use in the prediction, b) checking the quality of the information and number of observations, and c) analysis of the auto-correlation of the historical observations.

Step 2. Estimation: a) determination of the estimates of the parameters, using the least squares criterion, b) application of an iterative procedure searching for a sum of squares function, previously specifying the preliminary estimates of the unknown parameters.

Step 3. Diagnostic check: a) adequacy test, done after the models were fitted to the data, b) analysis of the difference between the observed and expected results, c) application of the Box-Pierce chi squared.

Step 4. Prediction: a) selection and design of the definitive model, b) data processing, c) prediction of the future values of the time series.

The resulting model for estimating the expected demand of diabetes was a model with average movement operator of order 1. To calculate financial consequences caused by changes in the epidemiological profile and the demand by type of institution, an inflationary index projected to 2010–2012 was developed and applied, based on the price index to consumers by the Bank of Mexico (*Banco de México*). Results are in US dollars, at the exchange rate of 1 = 13.35, corresponding to January, 2012 [29].

## Results

After making the projection of expected cases for the 2010–2012 period, we decided to take the year 2011 as a cut-off point to determine the costs of care for diabetes mellitus for each sector of the health system. The national annual average cost of case management was $ 707 US dollars and the base population estimate was 4,854,689 patients diagnosed with diabetes and treated in different institutions of the health system.The direct costs represent 45% of the total costs of diabetes mellitus in Mexico. Table 1 shows the distribution of the direct costs among the main items of economic impact in the management of diabetes for different institutions of the health system.

**Table 1:** Direct, indirect and total costs attributable to the diabetes mellitus to 2011 in México: SSA, IMSS, ISSSTE, users, and private health insurance (in US dollars).

Costs	Healthcare service provider					Total
	SSA	IMSS	ISSSTE	Users pocket	PHI	
DIRECT COSTS						
Consultations/diagnosis	71,011,135	160,290,894	37,503,003	310,619,140	17,920,329	597,344,501
Drugs	158,133,310	357,498,753	83,514,756	692,347,435	39,943,108	1,331,437,362
Hospitalisation	47,476,705	107,167,486	25,073,817	207,674,140	11,981,182	399,373,330
Retinopathy	14,437,970	32,590,336	7,625,104	45,930,958	2,649,862	*103,234,230*
Cardiovascular disease	13,125,455	29,627,576	661,913	80,379,150	4,637,260	128,431,354
Nephropathy	95,815,653	216,281,301	50,602,990	430,602,624	24,842,443	818,145,011
Neuropathy	4,725,155	10,665,924	2,495,485	9,186,191	529,973	27,602,728
Peripheral vascular disease	3,150,100	7,110,616	1,663,655	8,037,924	463,730	20,426,025
Total direct	407,875,483	921,232,886	209,140,723	1,784,777,562	102,967,887	3,425,994,541
INDIRECT COSTS						
Mortality	22,676,240	53,267,038	12,170,707	108,116,320	na	196,230,305
Permanent disability	471,886,615	1,108,472,727	253,269,190	2,258,429,948	na	4,092,058,480
** **Temporary disability	7,123,953	1,673,432	3,823,530	3,603,879	na	16,224,794
Total indirect	501,686,808	1,163,413,197	269,263,427	2,370,150,147	na	4,304,513,579
Total costs	909,562,291	2,084,646,083	478,404,150	4154,927,709	102,967,888	7730,508,120

Source: Arredondo et al. (2012) Costos y consecuencias financieras del cambio en el perfil epidemiológico en México. INSP–Update of probabilistic models, January 2012.

Exchange rate: January 2012, 1 US$ = 13.35 Mexican $.

95% CIs. Box–Pierce statistical test (p<0.05).

IMSS, Mexican Institute for Social Security; ISSSTE, Institute for Social Security and Services for State Workers; na, not available; PHI, private health insurance; SSA, Ministry of Health.

Direct costs represent 44% of the total costs of diabetes mellitus in Mexico (see table 1). The distribution of direct costs among the main items of economic impact in the management of diabetes for different institutions of the health system and to users. With respect to the cost of the different functions of production, it is worth noting that the inputs with the greater impact refer to medicines, followed by costs of outpatient services and to a lesser degree, by costs of hospitalization in cases of acute complications due to diabetes mellitus, without considering the management of chronic complications.

For the relative weight of the cost in the overall management of the main complications of diabetes, in all the institutions, the greater impact is in the costs for managing diabetic nephropathy, followed by–from largest to smallest–the costs of retinopathy, cardiovascular disease, diabetic neuropathy and finally, peripheral vascular disease.

With respect to the relative weight of the economic impact per origin of the costs, of the total direct costs, the greater economic impact corresponds to the pocket of the health service users, that is to say, for each 100 dollars that are spent in diabetes in Mexico, 51 come from the users’ pockets (private sector health expenditure). Followed by -in order of importance by their relative weight- the IMSS, the SSA and the ISSSTE. With respect to the economic impact of diabetes among the different health institutions of the public sector, it is important to point out that the economic impact for the IMSS is more than double that of the SSA and four times more than that the ISSSTE. The same tendencies were observed when determining the costs of diabetic complications. With regards to the indirect costs of diabetes mellitus health care, in this dimension of costs, we were only able to estimate the indirect costs for users that sought care in the three main institutions of the public sector. These costs represent 56% of the total cost of diabetes mellitus in Mexico. They are distributed in 3 categories of estimation: costs by premature mortality (5%), costs by permanent handicap (93%) and costs by temporary handicap (2%).

With respect to the costs of case-management in the three studied institutions, the results show important differences among the institutions and among the hospitalized case management and outpatient cases. Before discussing the details of the results on the economic evaluation, it is important to mention that all the results on costs were expressed in U.S. dollars for an international comparison. From these findings it is possible to establish minimum and maximum ranges by type of disease for the three main institutions of the health system in Mexico. Indeed, on the cost of case-management of hospitalized patients, the results are in a range going from $613.71 to $887.14 (in U.S. dollars). The smallest cost corresponded to the SSA and the greatest cost to the IMSS. The same tendencies are observed for the cost of case-management of outpatient cases.

The differences between costs of case-management and total costs attributable to diabetes in Mexico, observed among the different studied institutions, are explained by the significant differences in the specific populations that demand services, by the cost of inputs and by the way in which these inputs are combined at the time of producing the demanded service. However, the quality of care provided by each institution is an intervening and determining variable which relates to the actual cost of diabetes care for each of the main health care institutions in Mexico. With respect to the effects of the observed epidemiological changes in health care demand for hospital and ambulatory services for the 2009–2011 period, the expected tendency is towards an increase in costs, although the increase is more relevant for the insured than for the uninsured population.

The insured population (IMSS and ISSSTE), makes the greatest use of financial resources. This increase is not only due to the increase in health care demand from the changes in the epidemiological profile, but also to the increase in the percentage rate of the projected inflationary index for the 2010–2012 period. It is important to point out that in the case of the uninsured population (SSA), it not only has the smallest financial requirement but also the tendencies of the required financial resources for this population are much more moderate that in the case of the insured population.

As far as the costs to the public sector, when comparing the results of the direct costs for the different health care models according to the type of institution, the IMSS health care model paid the greatest cost, followed by SSA and finally ISSSTE with the smallest direct cost. The same type of tendency was observed for the case of the direct costs of the ambulatory health care model. With respect to the indirect costs by type of disease it is important to point out that the obtained cost is the minimum amount that would appear in each type of institution in agreement with the demand for the year 2011 as a cut-off point.

On the costs to users’ pockets, it is noteworthy that the high relative weight of the source of expenses for diabetes is from the family income; this has implications in terms of equity and access to health care in Mexico. Indeed, we can say that from each 100 dollars that are spent in diabetes in Mexico, approximately 51 come from the household/family income. This represents a social burden of very high impact that evidently will have a considerable effect on the measurement of catastrophic health expenditures in the country, more importantly because it is a very expensive disease and one of high priority as a public health problem in Mexico.

With respect to the indirect costs, although they do not constitute a direct impact on the health budget, in terms of cost and social impact, they do represent a high burden that society in general will have to assume, mainly in terms of lost productivity by premature death and disability whether it is temporary or permanent.

## Discussion

First of all, it is important to note that the results presented here, differ substantially from other results on diabetes costs, which have been published and they come from the same project. Indeed, the validation of the analysis model to study the economic burden of diabetes in Mexico, as a pilot, generated the first partial results in 2009 [30]. Such partial results published in 2009, used the same model of analysis, but only referred to a probabilistic estimate corresponds to 483 000 from diabetic population that would be expected for the year 2010 and only to one region of the country, resulting in a partial cost $778 427 475.

The new results represent the total economic burden for 100% of diabetic patients were treated in 2011 across the country (4,854,689), in different institutions of the health system. So while the economic burden partial data published in 2009 was U.S. $778,427,475, the total economic burden of the data presented here was 7,730,508,120. This substantial difference is one of the reasons why in this manuscript we highlights health disparities that represents the economic and epidemiological burden of diabetes for the total population of diabetics in Mexico, both for patients and the health system.

The relevance of incorporating economic and epidemiological aspects in the clinical perspective constitutes a comprehensive proposal for the analysis health disparities and evaluation of the performance of the health system in the context of health reforms. Indeed, the results of health systems research with an economic, clinical and epidemiological perspective becomes relevant in view of the evidence presented in this study.

First of all, as some of the health reform projects progress, the cost of health care to hospitalized patients with chronic-degenerative diseases will be higher than the cost of health care for hospitalized and ambulatory cases of infectious disease; therefore, the greater change with regards to the epidemiological transition will be observed in terms of greater financial consequences in the production of health care services for future health care demands of chronic-degenerative diseases, particularly in the case of diabetes mellitus.

The observed and expected changes in the epidemiological profile of diabetes, both in the public sector and in the pocket of health care users, will lead to a financial competition for resources. This will be in such a way that financial resource allocation for the demand or production of health services directed to diabetes will be affected by the production or demand of services for infectious diseases and other chronic diseases like hypertension. In this sense, the internal competition in the use and allocation of economic resources requires knowledge of the approximate financial requirements to produce the services that will be demanded in the short and medium terms. Therefore, the production and financing of health services for diabetes will require the incorporation of clinical, economic and epidemiological indicators, integrating them under the efficiency criterion.

If the risk factors and the different health care models for ambulatory and hospital care remained more or less as they are at the moment in the three studied institutions, the financial consequences of diabetes would be of greater impact for the IMSS, followed in order of relevance by the SSA and ISSSTE. On the other hand, the financial requirements for the treatment of diabetes, both in ambulatory and hospital services would represent approximately 9% of the total budget assigned to the uninsured population and approximately 16.5% of the assigned total budget for the insured population.

Diabetes care in Mexico is distributed more or less in the following way: 48% of the population is taken care of by social security institutions; 42% by institutions that serve the uninsured; and 10% of the population uses private care institutions. According to our results, this means that, with respect to direct costs, out of each 100 dollars that are spent in diabetes in Mexico, 51 are spent on 10% of the population, 34 on 48% (insured) of the population and 15 dollars on the remaining 42% of the population (uninsured). In terms of health disparities, these data show clearly that there is a problem related to equity and poor health care accessibility in the different sectors of the Mexican population.

It is quite evident that the problem of diabetes in Mexico not only represents a high economic impact from the perspective of all the epidemiological, economic and organizational considerations that were mentioned in the introduction of this text, but there are also problems of inequity and health care accessibility according to the social group to which the patients with diabetes and their families belong to.

### Conclusion

The disparities of the economic burden of diabetes in Mexico show a number of implications for health systems, families and patients to be taken into account in the context of increasing trends of diabetes and its complications.

In this regard we would like to highlight a list of suggestions and lessons from the disparities caused by the economic burden on non-communicable diseases should be taken into account in the resolution of the challenges of diabetes for middle-income countries:

1. The evidence on changes in costs and in the demand for health care in diabetes patients can be used as a reference for the allocation of resources directed towards diabetes by different types of public institutions. With a knowledge of likely financial requirements, each institution could then target, effectively and efficiently, the necessary resources for promotion, prevention, healing and rehabilitation.
2. A consequence of the implementation of cost-monitoring systems is the design and application of strategies for cost-containment for weight-by-cost items. For example, knowing that the cost of medicines is high, it will be necessary for each institution to review its agreements with the pharmaceutical industry on the consolidated buying of medicines for diabetes.
3. The development of economic indicators would enable the design of resource allocation patterns based on efficiency criteria with regard to clinical, epidemiological, economic and administrative aspects. Each institution could develop models for the distribution of resources in accordance with the changes in costs and epidemiological factors expected in future years.
4. As a ‘Citizen Observatory of Diabetes’, social organizations could suggest and develop follow-up programs for the costs of diabetes in different public and private health institutions. The Observatory should function as a monitoring system that would see how much is being spent on diabetes management and what the money is being spent on.
5. Knowledge of the relative weight of diabetes management, based on the annual family income, as well as precise knowledge of the cost of complications to the users, should be made available through a bulletin sent to diabetic patients and their relatives, and to the community as a whole. Knowledge of the high costs of diabetes per family could lead to a greater self-awareness, as well as to efforts in avoiding complications caused by the disease.
6. A list of recommendations is needed to promote greater self-care, control of risk factors and awareness of the benefits of carrying out these measures; more importantly, it would help diabetic patients avoid falling into a catastrophic cost situation (which could mean an impact of >30% of the family income).
7. Within the field of the health services, and in research and development in human resources, our results allow us to put a greater emphasis on health evaluation and promotion, with important changes in the social aspects of diabetes as a high-priority public health problem.
8. The treatment of diabetes should be approached from an integrated perspective: clinical, economic, epidemiological and organizational. In other words, an integrated approach to the problem of diabetes requires the development of indicators of clinical and economic efficiency, expected epidemiological changes and demands for health care services.
9. The economic implications of diabetes let us see which is closely related to social disparities, especially in contexts of great social inequality ((particularly in low and middle income countries). In this sense, the analysis of the economic burden of diabetes must be accompanied by analysis of other social determinants of diabetes, so that it can be identified when diabetes and its complications are a cause or effect of social disparity and consequently of a disparity in health.
10. Health disparities generated by the economic burden of diabetes suggests the need to design and review the current organization of health systems and the relevance of moving from biomedical models and curative health care to preventive and socio-medical models to meet expected challenges from diseases like diabetes in middle-income countries.
